# Pulsatile *ex vivo *perfusion of human saphenous vein grafts under controlled pressure conditions increases MMP-2 expression

**DOI:** 10.1186/1475-925X-10-62

**Published:** 2011-07-21

**Authors:** Sara Dummler, Stefan Eichhorn, Christian Tesche, Ulrich Schreiber, Bernhard Voss, Marcus-André Deutsch, Hans Hauner, Harald Lahm, Rüdiger Lange, Markus Krane

**Affiliations:** 1 German Heart Center Munich at the Technische Universität München, Department of Cardiovascular Surgery, Lazarettstrasse 36, D-80636 Munich, Germany; 2 Technische Universität München, Research Center for Nutrition and Food Science, Nutritional Medicine Unit, Gregor-Mendel-Strasse 2, 85350 Freising-Weihenstephan, Germany

**Keywords:** Atherosclerosis, Bypass-Surgery, MMP, Perfusion, Pulsatile flow, vein graft

## Abstract

**Background:**

The use of human saphenous vein grafts (HSVGs) as a bypass conduit is a standard procedure in the treatment of coronary artery disease while their early occlusion remains a major problem.

**Methods:**

We have developed an *ex vivo *perfusion system, which uses standardized and strictly controlled hemodynamic parameters for the pulsatile and non-static perfusion of HSVGs to guarantee a reliable analysis of molecular parameters under different pressure conditions. Cell viability of HSVGs (n = 12) was determined by the metabolic conversion of 3-(4,5-dimethylthiazol-2-yl)-2,5-diphenyl-tetrazolium bromide (MTT) into a purple formazan dye.

**Results:**

Under physiological flow rates (10 mmHg) HSVGs remained viable for two weeks. Their exposure to arterial conditions (100 mmHg) was possible for one week without important reduction in viability. Baseline expression of matrix metalloproteinase-2 (MMP-2) after venous perfusion (2.2 ± 0.5, n = 5) was strongly up-regulated after exposure to arterial conditions for three days (19.8 ± 4.3) or five days (23.9 ± 6.1, p < 0.05). Zymographic analyses confirmed this increase on the protein level. Our results suggest that expression and activity of MMP-2 are strongly increased after exposure of HSVGs to arterial hemodynamic conditions compared to physiological conditions.

**Conclusion:**

Therefore, our system might be helpful to more precisely understand the molecular mechanisms leading to an early failure of HSVGs.

## Background

Coronary artery bypass grafting (CABG) using venous grafts is a standard procedure in the treatment of advanced coronary artery disease. However, vein graft occlusion implanted in an arterial pressure environment is still a major problem [1]. Approximately 15 to 20% of human saphenous vein grafts (HSVGs) occlude within one month and 25% within the first year. Ten years after CABG about 50% of the HSVGs are occluded and 25% have been severely stenosed [2-6]. Early changes in vein grafts include endothelial disruption leaving the graft vulnerable to thrombotic incidents and smooth muscle cell (SMC) migration and proliferation from the media into the intima within the first week after grafting [1,7]. The vein graft intimal thickening and remodeling occurs as an adaptation to increased wall stress and arterial flow with up to 15% of graft stenosis during the first year [8]. Under physiological conditions human saphenous veins are exposed to low pressure conditions (~5-10 mmHg), a nonpulsatile flow and a shear stress of 1-6 dyne/cm^2 ^[9]. After grafting and implantation into the coronary artery system the graft must support higher pressure conditions (~60-140 mmHg), a pulsatile flow and a shear stress range of 10-70 dyne/cm^2 ^during the cardiac cycle [9,10]. Beyond the first year after bypass surgery the development of graft atheroma and accordingly atherosclerotic vein graft stenosis is the dominant process underlying the failure of HSVGs [1,11]. Formation and evolution of atherosclerotic plaques are associated with variations in matrix metalloproteinase (MMP) expression. The gelatinases play a central role in matrix degeneration and SMC migration, a process which substantially contributes to vein graft failure. The involvement of different MMPs in vascular remodeling has been shown [12-14] whereas little is known about the specific role of gelatinases in HSVGs. While MMP-2 is either absent or only present at low levels in normal veins, its expression becomes elevated after graft implantation which may be a response to injuries during graft preparation or the exposure to the arterial environment [8]. It is generally accepted that the arterial mechanical environment plays a role in vein graft failure, yet the specific mechanical conditions and biological mechanisms have not been completely understood. Vessels cultured under static conditions have been widely used to study effects of pre-existing intimal hyperplasia (IH) [15]. Berceli et al. used a rabbit model to analyze intimal changes and MMP gene and protein expression after bilateral common carotid interposition vein grafting with defined regions of different wall shear [16]. The group of Patterson has used HSVGs in organ culture under static conditions or perfusion for seven days with the restriction of shear force calculation and the differentiation just between low-flow and high-flow conditions [17]. Compared to the animal model of Berceli et al. the *ex vivo *perfusion system of Patterson et al. has a nonpulsatile hemodynamic environment, no blood-surface interaction and potential problems with delivery of nutrition or gas. Gusic and colleagues investigated the role of the mechanical environment in vein remodeling in a higher developed *ex vivo *perfusion system with a main focus on medial and intimal growth in the perfused veins. They ran their perfusions system with five different *ex vivo *hemodynamic environments and showed that pressure and shear stress act independently to regulate vein remodeling [7]. Yet, their study had the limitation of unstable pressure profiles during the course of the experiment. In the present study we have developed an *ex vivo *perfusion system which can be used to perfuse HSVGs with tightly controlled, steady and standardized perfusion profiles. We have defined the viability time course of perfused HSVGs exposed to arterial and venous perfusion profiles. In addition, we provide evidence that our system is suitable to detect alterations of molecular markers such as MMP-2 as a consequence of preparative injury or increased arterial perfusion pressure.

## Methods

### Tissue Preparation

Nonvaricose HSVGs were obtained from 35 patients (mean age 71.4 ± 7.7 years; nine females, 26 males) undergoing CABG surgery in the German Heart Center Munich. The endoscopically harvested vein grafts were kept in autologous blood at room temperature until implant. One part of the graft was immediately stored in Ringer solution on ice and transferred from the operating room to the laboratory. One small piece was directly snap-frozen in liquid nitrogen and stored at -80°C until further use as unperfused control tissue. This piece served as a reference to determine relative gene expression. The other part of the vein was mounted into the perfusion device as described. The procedure was acknowledged by the local ethical committee (Ethikkommission der TU München, Project no. 1588/06).

### Ex vivo perfusion system

The circuit of the perfusion system is driven by a roller pump ISMATEC S2 (Wertheim, Germany) producing a pulsatile and non-static flow. All silicon tubings and the vessel chamber are sterilized prior to use. The vessel mounting procedure is carried out under a biological safety cabinet (NuAire, Plymouth, MN). Constant pressure conditions are maintained using a syringe pump (MC Medizintechnik GmbH, Alzenau, Germany). The entire system is placed into a styrofoam-isolated chamber to maintain a constant temperature of 37°C. Disposable pressure sensors (DPT-9300, Codan Critical Care, Forstinning, Germany) are placed on both sides of the vessel chamber to permanently monitor and facilitate the control of pressure conditions of the circuit. All functions and settings are controlled by a PC with a program written in java. Pressure is controlled by a PID-algorithm, data are logged continuously.

### Perfusion of human saphenous vein grafts

HSVGs were fixed in the perfusion device by suture ligation (Ethibond Vicryl 3-0, Ethicon GmbH, Norderstedt, Germany) and adjusted to a length matching the *in vivo *conditions. Total time from operating room to perfusion was less than one hour. The perfusion medium was DMEM/Ham's F-12 (PAA, Marburg, Germany) supplemented with 10% FCS, 2 mM glutamine and antibiotics (100 U/ml penicillin and 100 μg/ml streptomycin). Veins were perfused with venous conditions (flow: 5 ml/min, 10 mmHg, n = 12) or with arterial conditions (flow: 50 ml/min, 100 mmHg, n = 12) for various time periods. At the end of each experiment vein ends were discarded. The other part of the vein was snap-frozen in liquid nitrogen and stored at -80°C until further use. In long-term experiments the medium was replaced every two days. The pH of the medium remained stable within this period.

### Determination of viability of vein grafts and histology

To verify tissue viability, a staining with MTT (Sigma, Munich, Germany) was performed. In the presence of metabolically active viable cells the yellow MTT is converted into a water-insoluble purple formazan product due to reduction by mitochondrial dehydrogenases and other cellular enzymes [18,19]. MTT was stored as a stock solution (5 mg/ml in PBS) at -20°C. Short segments of veins (n = 12) were incubated in MTT diluted in serum-free medium to 0.5 mg/ml for one hour at 37°C. To analyze potential degenerative changes in perfused vessels, sections of formalin fixed and paraffin-embedded samples were analyzed after a conventional hematoxylin/eosin staining.

### Quantitative RT-PCR analysis

Frozen tissue pieces were minced using a Precellys24 lysis and homogenization system (Peqlab, Erlangen, Germany) and total RNA was extracted using Trifast according to the manufacturer's recommendation (Peqlab). All RNA preparations were digested with DNase I prior to cDNA synthesis using Omniscript RT kit (Qiagen, Hilden, Germany). One μl of cDNA was amplified on a LightCycler 1.5 thermo cycler (Roche Diagnostics, Mannheim, Germany) using the QuantiTect SYBR Green Kit (Qiagen) and BSA (0.5 mg/ml) in a final volume of 20 μl. All primers were used in a final concentration of 0.5 μM. The following primers were used: β-actin forward 5' CCA ACC GCG AGA AGA TGA 3', β-actin reverse 5' CCA GAG GCG TAC AGG GAT AG 3', MMP-2 forward 5' TGC TGG AGA CAA ATT CTG GA 3', MMP-2 reverse 5' GAT GGC ATT CCA GGC ATC 3'. They amplify fragments of 96 and 90 bp, respectively. After an initial activation of *Taq *polymerase for 15 min at 95°C specific products were amplified during 40 cycles using the following conditions: 15 sec at 94°C (denaturation), 20 sec at 60°C (annealing) and 20 sec at 72°C (elongation). The relative expression levels of MMP-2 in individual samples were calculated in relation to the expression of the β-actin housekeeping gene. To compare independent samples the ratios of MMP-2/β-actin were calculated.

### Zymography

MMP-2 protein activities were evaluated by a standard gelatine zymography. Briefly, 100 mg of frozen HSVG tissue were homogenized in ice cold zymogram buffer (150 mM NaCl, 1 μm ZnCl_2_, 1.5 mM NaN_3_, 20 mM CaCl_2_, 0.01% Triton X-100, 10 mM cacodylic acid, pH 5.0). Samples were centrifuged at 4°C for 10 min at 20.000 × *g*. The supernatant containing proteins was removed and stored at -80°C until further use. Ten μg of extracted protein were mixed with zymogram loading buffer (62.5 mM Tris/HCl, pH 6.8, 25% glycerol, 4% SDS, 0.01% bromophenol blue) and separated in 15% SDS-PAGE gels containing 1 mg/ml type A gelatine from porcine skin (SIGMA-Aldrich, Taufkirchen, Germany). To renature proteins, gels were washed two times in 2.5% Triton X-100 for 15 min at room temperature and subsequently incubated in developing buffer, pH 7.5 (200 mM NaCl, 50 mM Tris, 5 mM CaCl_2_, 0.02% Brij-35) overnight at 37°C. Gels were stained with 0.5% Coomassie Blue R250 in 40% methanol/10% acetic acid for 15 min and destained in 40% methanol/10% acetic acid until clear bands of lytic activity appeared. The reaction was stopped by transfer of gels in aqua bidest. Gelatinolytic activity was quantified using ImageJ software (version 1.43 u, National Institute of Health). The pixel intensities of bands within each gel were normalized against the respective control of unperfused venous tissue.

### Statistical analysis

For the analysis of gene expression levels and MMP-2 gelatinolytic activity the comparison was made using the unpaired Student's *t*-test. Differences in the vessel viability were calculated using the Mann-Whitney U-Test. Differences were considered to be significant at values of *p *< 0.05.

## Results

### Establishment of the ex vivo perfusion system

**Figure 1:**
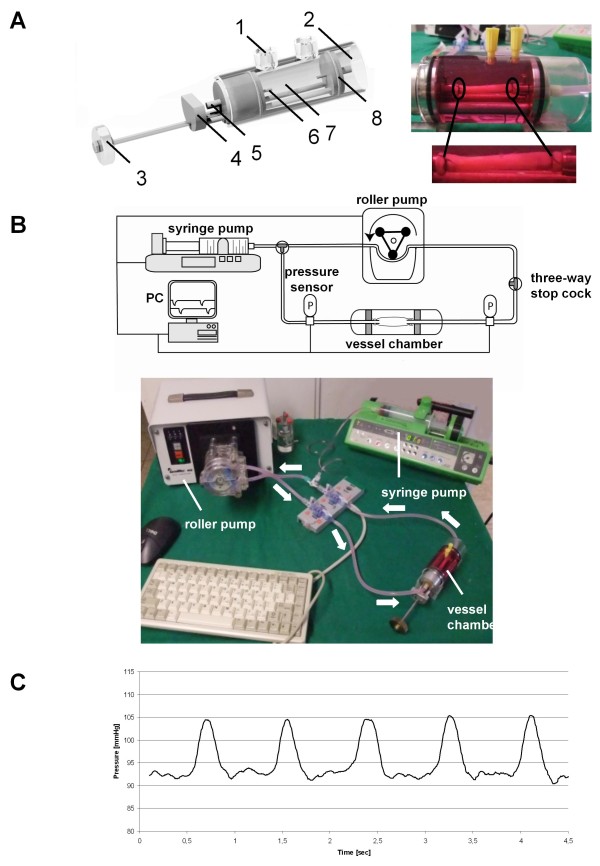
***Ex vivo *perfusion system**. **A) **Vessel chamber. 1: ports for medium exchange, 2: glass tubing, 3: rotating wheel for length adjustment of sliding unit, 4: sliding unit, 5: Luer connector to circuit, 6: tapered adapter to fix veins, 7: vessel chamber, 8: sealing ring. **B) **Components of the perfusion system. **C) **Logged pressure time profile.

Twenty four veins from twenty three patients were used for the *ex vivo *perfusion experiments to establish and proof the reliability of the system. The veins were fixed on tapered conical metal adapters with circular striae to ensure a tight fit of the grafts throughout the whole experiment (Figure 1A). All components used in the vessel chamber are biocompatible (PEEK, 316 L) thereby avoiding any potential interactions with the veins. The grafts were brought to their initial length using the adjustment device. Deaeration was performed by using two three way stop cocks. An overview showing the components of the perfusion system is given in Figure 1B. Under arterial pulsatile (Figure 1C) and non-static flow conditions three veins were cultured for one day, five veins for three days and four veins for five days. To establish the reliability of the system we perfused five HSVGs for one, three veins for three and four veins for five days with low pressure conditions (10 mmHg, flow rate 5 ml/min) which mimics the physiological venous pressure profile. Sensors on both side of the vessel chamber permanently surveyed the pressure inside the circuit (Figure 1B). In case of a pressure decrease a tiny volume of medium was injected into the circuit from an external medium reservoir mounted in a syringe pump. With this setup we were able to maintain the pressure constantly within a deviation of less than 2 mmHg during the whole experiment. The perfusion conditions were controlled by a customized software package. By using a PID control algorithm to control the syringe pump a constant pressure could be secured throughout the whole experiment. Pressure data were logged every 10 seconds and were analyzed after every trial.

### Human saphenous veins support arterial perfusion conditions for one week

**Figure 2:**
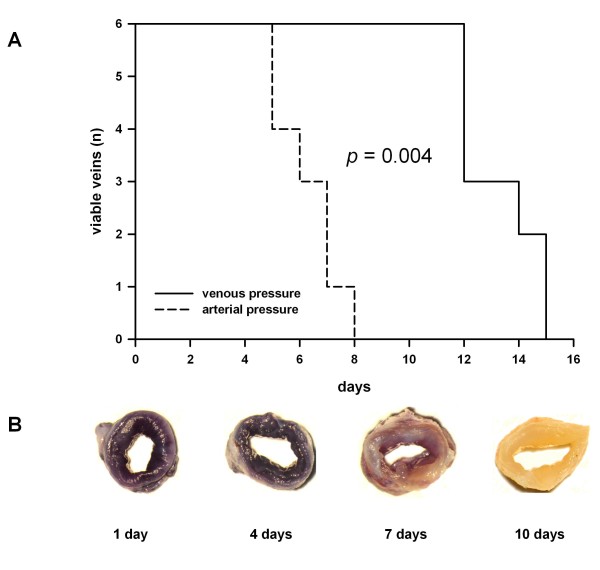
**Viability of human saphenous vein grafts under different pressure conditions**. **A) **Veins were perfused with either venous (10 mmHg) or arterial pressure profiles (100 mmHg). Viability of cells was tested using the MTT conversion assay as described in Material and Methods. *n *= 6 for both conditions. **B) **Representative MTT stainings of HSVGs at different time points.

Under venous conditions all tested veins contained viable cells throughout the vessel wall for up to 12 days indicated by a conversion of MTT into a purple formazan product (data not shown). Thereafter, the viability dropped (Figure 2A). We then analyzed to what extent the veins would support an elevated pressure which corresponds to the arterial situation (100 mmHg, flow rate 50 ml/min). After one and four days of arterial perfusion all veins were fully viable (Figure 2B) and showed an intensive purple staining. Even after seven days the cells clearly showed metabolic activity though to a reduced degree (Figure 2B). Beyond one week the veins did not support these elevated pressure conditions evidenced by the complete lack of MTT conversion (Figure 2B). Thus, we have successfully established a standardized system, which allows the perfusion of human veins with an arterial pressure profile for up to one week.

To further explore potential pathological changes in HSVGs upon perfusion, we investigated tissue sections from veins after perfusion with venous or arterial pressure profiles at different time points by a hematoxylin/eosin staining. As a reference, we used an unperfused section of the same vein. Exposure to venous pressure for three days did not change the histology and even after five days a minor thickening of the intimal layer was evident (Additional File 1, Figure S1). After arterial perfusion for one day also no major changes could be noticed. However, after three days the intimal layer started to visibly thicken and after five days extensive hyperproliferative areas were seen (Additional File 1, Figure S1).

### Arterial perfusion conditions up-regulate MMP-2 gene and protein expression

**Figure 3:**
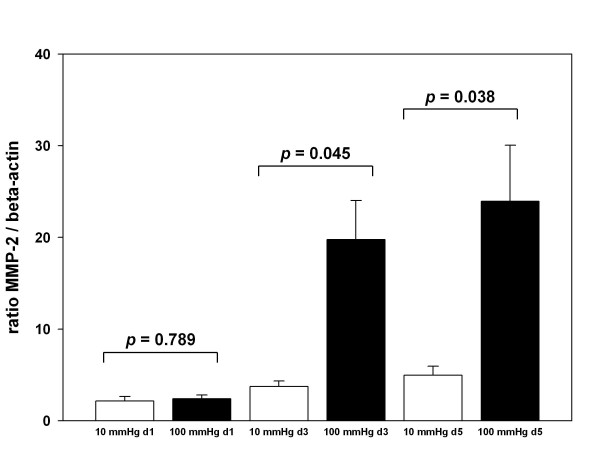
**Increase of MMP-2 expression in human saphenous vein grafts after exposure to arterial pressure**. HSVGs were perfused with venous (10 mmHg, white bar) or arterial (100 mmHg, black bars) pressure profiles for the indicated time relative to the unperfused controls of the same veins, respectively. 10 mmHg 1 day (*n *= 5), 10 mmHg 3 days (*n *= 3), 10 mmHg 5 days (*n *= 4), 100 mmHg 1 day (*n *= 3), 100 mmHg 3 days (*n *= 5), 100 mmHg 5 days (*n *= 4). The MMP-2/β-actin ratio in unperfused veins was arbitrarily set to 1.

**Figure 4:**
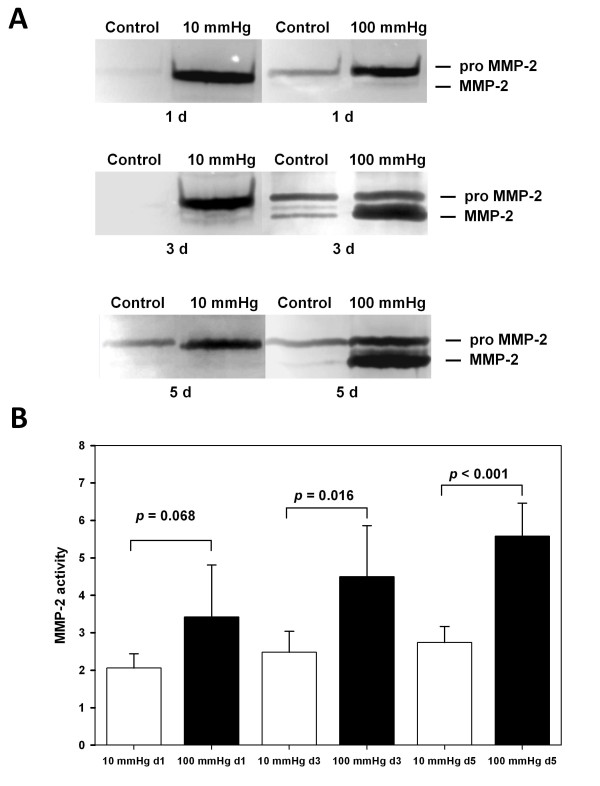
**Zymographic analysis of MMP-2 activity**. **A **Representative results are shown for HSVGs perfused with venous or arterial pressure. Controls represent MMP-2 activity of unperfused tissue taken from the same vein (n = 5 for each condition). **B **Quantitative analysis of MMP-2 gelatinolytic activity. The activity in unperfused veins was arbitrarily set to 1.

We next addressed the question whether the system is suitable to record alterations in gene expression as a consequence of exposure to different pressure profiles. To that end we analyzed MMP-2 as its expression is known to increase as a consequence of hypertension and vein graft preparative injury [20-22]. We first determined MMP-2 expression in human veins which were perfused with 10 mmHg for one day which revealed a baseline ratio of MMP-2/β-actin of 2.2 ± 0.5 (n = 5) compared to unperfused control tissue (Figure 3). Extended perfusion of HSVGs for three days gave a similar result (3.7 ± 0.6, n = 3) and perfusion for five days under venous conditions showed a slightly increased gene expression of 5.0 ± 1.0 (n = 4) (Figure 3). No significant difference could be observed between venous perfusion of HSVGs for one or three days. Perfusion with 10 mmHg revealed statistical significance between five days and one day (p < 0.05) (data not shown), probably due to the elongated exposure in the *ex vivo *system. Perfusion of HSVGs with 100 mmHg for one day yielded an MMP-2 gene expression ratio which was similar to the reference (2.4 ± 0.4; n = 3) (Figure 3). However, MMP-2 gene expression was significantly up-regulated when HSVGs were exposed to an arterial perfusion profile for three days (19.8 ± 4.3; n = 5). This value increased further when arterial conditions were extended to five days (23.9 ± 6.1; n = 4) (p < 0.05; Figure 3). Thus, the elevation of MMP-2 gene expression starts rapidly when HSVGs are exposed to arterial flow conditions and it is maintained at this high level for at least five days. We then determined whether this change in RNA expression was also reflected on the protein level in a zymographic analysis. Under venous pressure MMP-2 activity corresponding to a molecular weight of 72 kD was detected, corresponding the activity of pro-MMP-2 (Figure 4A). Exposure to an arterial pressure for one day yielded similar patterns (Figure 4A). However, when arterial pressure profiles were applied for three or five days gelatinolytic activities were strongly increased. In particular, the 63 kD form of MMP-2 showed a heavily increased activity when compared to unperfused control tissues (Figure 4A). Quantification of the gelatinolytic activity confirmed our results of MMP-2 mRNA expression (Figure 4B). Gelatinase activity did not increase significantly between venous (2.1 ± 0.4, n = 5) and arterial (3.4 ± 1.4, n = 5) perfusion after one day. According to the results of mRNA expression extended perfusion with arterial pressure for three (4.5 ± 1.4, n = 5) or five days (5.6 ± 0.9, n = 5) revealed significantly elevated MMP-2 gelatinolytic activity compared to venous conditions (2.5 ± 0.6, n = 5 and 2.7 ± 0.4, n = 5, respectively).

Thus, our novel *ex vivo *perfusion system proved its ability to monitor alterations in the expression of genes which are expected to increase their activity due to elevated pressure conditions on the RNA and protein level.

## Discussion

A major problem with HSVGs remains their occlusion after a certain time. Transposition of a vein segment and exposure to the arterial hemodynamic environment leads to an acute increase in flow rates and intraluminal pressure and is thought to be a potential trigger for the pathological remodeling of HSVGs [22,23]. Gene expression profiling approaches revealed that many genes and multiple pathways are differentially regulated under these conditions [24-26]. In the present study, we have established an *ex vivo *perfusion system developed to mimic the arterialization of HSVGs. The culture system provides the ability to reproduce the initial events taking place when the grafted vein is exposed to arterial hemodynamic conditions. Therefore, our system may represent a valuable and reasonable approach to identify molecular mechanism underlying the early stages of bypass grafting. Several *in vitro *and *in vivo *studies have demonstrated changes in graft morphology, viability, cellular density or gene expression under arterial conditions [7,16,22,27,28]. Saucy et al. for instance used an *ex vivo *vein support system to perfuse HSVGs with arterial conditions regarding shear stress, flow rate and pressure during a period of 7 and 14 days. They found significant IH and a marked increase in plasminogen activator inhibitor 1 (PAI-1) expression in the human veins after 7 and 14 days of perfusion [29]. A mathematical model of early vein graft IH induced by shear stress and based on experimental data with bilateral rabbit carotid vein grafts describes the general behavior of the remodeling process [23]. The group of Porter et al. demonstrated that arterial shear stress inhibits the development of IH in cultured vein pieces [30]. Previous studies have shown that SMC proliferation and migration depend on the activity of matrix-degrading enzymes. In fact, MMP-2 is an enzyme which is directly involved in vascular remodeling [14] and rodent animal models confirm that MMP-2 levels are increased under hypertensive conditions [20,21].

Within three days of perfusion under arterial pressure conditions in our perfusion system the expression of MMP-2 increased more than nine-fold and reached an even higher value after five days, similar to the activation of PAI-1 [31]. Our data are further supported by other reports which shows an increased *de novo *synthesis of MMP-2 in HSVGs perfused with artertial conditions [22] or in animal models who underwent vein grafting [8,16]. Berceli et al used a rabbit model with bilateral common carotid interposition vein grafting. They could show that accelerated IH resulting from reduction in wall shear stress was associated with an increase in MMP-2, mainly in an active form [16]. Our zymographic analyses are in accordance with their results and those of Patterson et al. [17], as we found strongly increased gelatinolytic activities in veins after perfusion with arterial pressure profiles particularly of the active form of MMP-2. As we compared HSVGs under venous or arterial pressure conditions, the elevation of MMP-2 can be attributed strongly to the arterial pressure profile. Both, gene and protein expression were significantly increased after perfusion with an arterial hemodynamic profile compared to venous conditions although all HSVGs had the same mechanical injuries after harvesting and mounting in the *ex vivo *perfusion system. Thus, the results of our perfusion system perfectly reflect the *in vivo *situation suggesting that genes which are involved in vascular remodeling are activated by arterial pressure. Therefore, our system can be used to analyze molecular parameters involved in such events in detail under standardized, tightly controlled and reproducible conditions.

An important advantage of our system is the possibility to mount vessels of variable length and diameter. The sliding unit allows a very flexible adjustment to guarantee that the vessel maintains its natural length and tension throughout the experiment. Our main focus was to setup an experimental system, which is suitable to reliably analyze molecular parameters as a function of altered pressure and flow conditions. Therefore, the most important point was to control the pressure conditions very stringently and also to keep them very stable. In pilot experiments we experienced a continuously decreasing pressure in the circuit, despite any leakage. Knowing that pressure affects gene expression such a behavior would be fatal for a desired molecular readout. With regard to this a unique feature of our perfusion system is the regulation of the mean pressure in the circuit by a computer controlled syringe pump. Decreasing pressure due to diffusion processes through out the silicone tubing [32] or relaxation of the vessel can be compensated automatically. Long time trials can be performed due to this amendment enabling an objectively constant mean pressure. In addition, up to four grafts can be perfused simultaneously within one circuit. Using MTT conversion we were able to confirm that HSVGs, which were perfused with a low-pressure profile in our system, remained viable for up to two weeks. This is in good agreement with other reports which have estimated the integrity of the vessels by histological or immunohistochemical methods [28,31,33]. Switching the conditions to an arterial pressure profile leads to a visible reduction of the MTT staining beyond five days of perfusion. These findings are similar to those of Miyakawa et al. who detected diminished cell viability in vein segments after perfusion with arterial conditions for four days [28]. They confirmed their results by hematoxylin staining which also reveals a reduction of nuclear staining on day four [28]. Gusic et al. could even show a dramatic increase in cell death index in all layers of the graft after one week [7]. We have also performed experiments in which HSVGs were perfused with pathologically elevated pressure (200 mmHg). However, under these conditions the grafts rapidly degenerated and after two days no MTT conversion was detected any more (data not shown). Our study, like others, is limited by the inability to perfuse the *ex vivo *system with autologous blood lacking blood cells, platelets, plasma, blood-surface interaction and the multitude of inflammatory and coagulation mediators playing an important role in the pathophysiology of IH development. However, because of technical reasons, we were not able to perfuse veins with blood by using a roller pump for perfusion to achieve a pulsatile flow. Platelets would be inevitably activated and blood cells destroyed during passage through the pump. Hemolysis could be avoided or highly reduced by using a centrifugal pump instead, which in turn produces a nonpulsatile flow. Inclusion of blood would provide exposure of the vein to a more physiological state, but may also confound the results with numerous other variables. Another limitation is the time-restricted viability of grafts in the *ex vivo *perfusion system which would not be prolonged by blood perfusion due to the accumulated metabolic waste products and inflammatory reactions. Despite these limitations, the findings of the current study highlight important potential in our understanding of the healing and adaptation of veins transplanted to the arterial environment. From the beginning of the development we tried to keep the total volume of the circuit relatively small. At present it comprises approximately 20 ml which is substantially lower compared to other systems which use volumes up to 500 ml [30,34]. If necessary the circuit can be scaled down even further to a volume of approximately 10 ml. Exogenous substances can be added in a defined concentration with a reasonable and affordable consumption of material, even during long-term experiments with repeated changes of medium and substances. One conceivable scenario is the induction of an inflammatory reaction in the vein followed by the addition of recently developed anti-inflammatory drugs [35,36]. Another most obvious application is the use of small molecules which have shown their anti-angiogenic potential *in vitro *[37]. Our system might unveil novel aspects about the activity of such molecules as the affected endothelial cells are located in their natural environment and maintain their physiological interactions with other cell types.

## Conclusions

In summary, we have developed a novel *ex vivo *perfusion system which maintains human veins viable for up to two weeks under a low pressure profile. The setup guarantees a tightly controlled and stable perfusion rate and the system proved to be suitable to record alterations in gene and protein expression induced by different perfusion profiles. Further advantages of our system are a total flexibility concerning the size of potential vessels and almost infinite possibilities in various research areas by the addition of defined amounts of exogenous substances into the circuit. Our *ex vivo *perfusion system and its applications may, therefore, help to improve the long-term patency of human bypass grafts.

## Competing interests

The authors declare that they have no competing interests.

## Authors' contributions

**- SD **prepared the human saphenous vein grafts and mounted them into the perfusion system, tested the viability of the vein grafts, performed quantitative RT-PCR-analysis and wrote the major part of the manuscript

**- SE **constructed the set-up of the perfusion system and helped with the development of the software

**- CT **helped to prepare and mount the HSVGs, participated in the quantitative RT-PCR-analysis and performed the zymographic studies

**- US **participated in the design and helped to build the perfusion device

**- BV **harvested the HSVGs for the perfusion system and viability experiments during CABG surgery and helped to draft the manuscript

**- MAD **participated in harvesting the HSVGs for the perfusion system and viability experiments during CABG surgery and helped to draft the manuscript

**- HH **participated in the design and coordination of the study and helped to draft the manuscript

**- HL **participated in the design of the study, performed statistical analyses and participated in writing of the manuscript

**- RL **harvested the HSVGs for the perfusion system, participated in the design of the study and its coordination and helped to draft and improve the manuscript

**- MK **initiated and designed major parts of the study, participated in coordination and writing of the manuscript

All authors read and approved the final manuscript.

## Supplementary Material

Additional File 1 **Figure S1**. Histological analysis of a representative formalin fixed and paraffin-embedded HSVG after perfusion with different pressure profiles and hematoxylin/eosin staining. The control represents the unperfused vessel. The other parts of the vein were perfused with physiological venous (10 mmHg) or arterial pressure (100 mmHg) for the time indicated. Click here for file
