# Supplementary figures and images for: Pulsatile ex vivo perfusion of human saphenous vein grafts under controlled pressure conditions increases MMP-2 expression
Source: Biomed Eng Online. 2011 Jul 21;10:62. doi: 10.1186/1475-925X-10-62 (PMC3148203; doi:10.1186/1475-925X-10-62)

## Slide 1
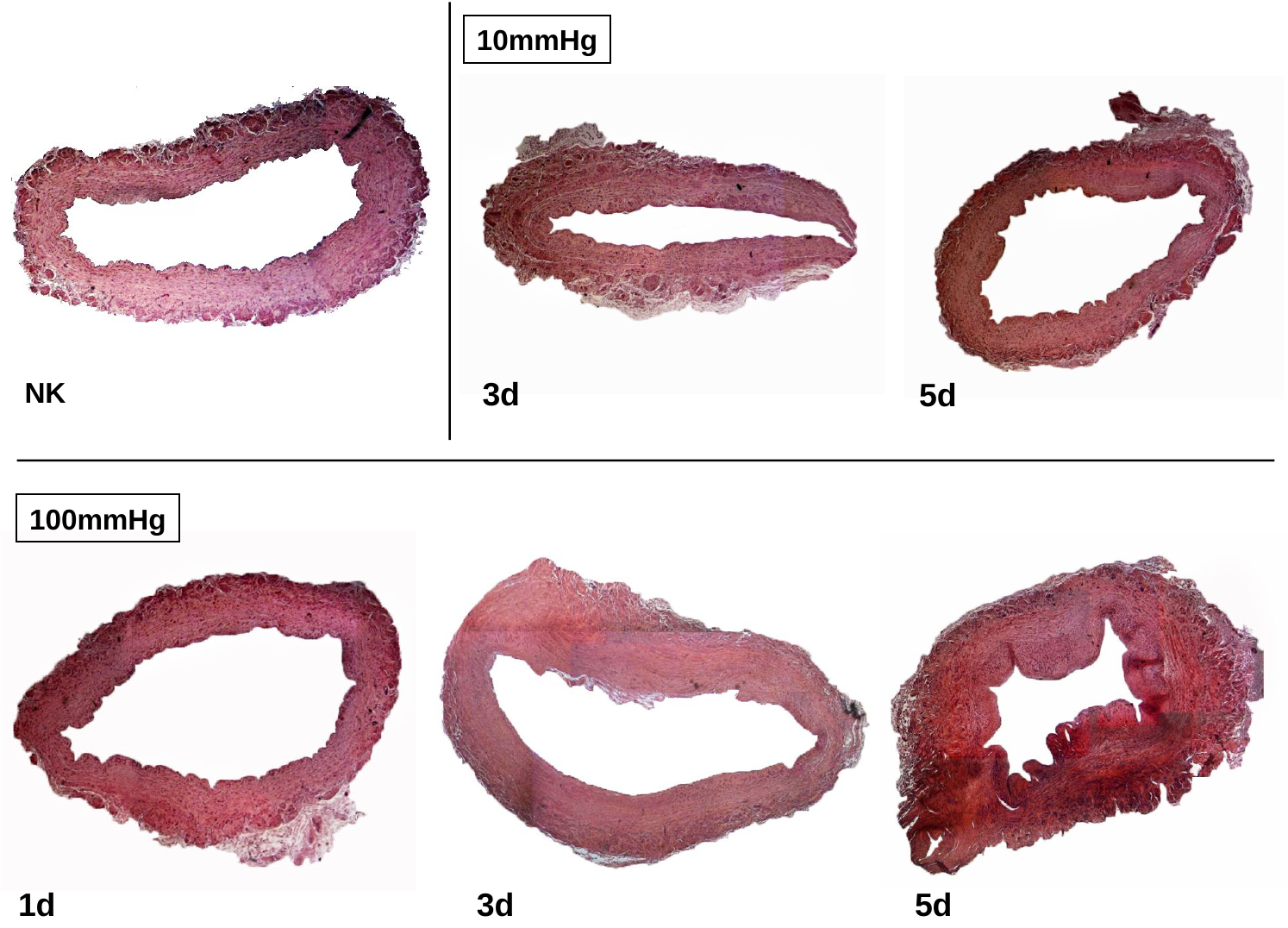

10mmHg
NK
3d
5d
100mmHg
1d
3d
5d

Supplement: Additional File 1 — Figure S1. Histological analysis of a representative formalin fixed and paraffin-embedded HSVG after perfusion with different pressure profiles and hematoxylin/eosin staining. The control represents the unperfused vessel. The other parts of the vein were perfused with physiological venous (10 mmHg) or arterial pressure (100 mmHg) for the time indicated. [file 1475-925X-10-62-S1.PPT]
